# Suicide among Young People and Adults in Ireland: Method Characteristics, Toxicological Analysis and Substance Abuse Histories Compared

**DOI:** 10.1371/journal.pone.0166881

**Published:** 2016-11-29

**Authors:** Ella Arensman, Marco Bennardi, Celine Larkin, Amanda Wall, Carmel McAuliffe, Jacklyn McCarthy, Eileen Williamson, Ivan J. Perry

**Affiliations:** 1 National Suicide Research Foundation, University College Cork, Cork, Ireland; 2 Department of Epidemiology and Public Health, University College Cork, Cork, Ireland; 3 St Patrick’s Mental Health Services, Cork, Ireland; University of New South Wales, AUSTRALIA

## Abstract

**Objective:**

Information on factors associated with suicide among young individuals in Ireland is limited. The aim of this study was to identify socio-demographic characteristics and circumstances of death associated with age among individuals who died by suicide.

**Methods:**

The study examined 121 consecutive suicides (2007–2012) occurring in the southern eastern part of Ireland (Cork city and county). Data were obtained from coroners, family informants, and health care professionals. A comparison was made between 15-24-year-old and 25-34-year-old individuals. Socio-demographic characteristics of the deceased, methods of suicide, history of alcohol and drug abuse, and findings from toxicological analysis of blood and urine samples taken at post mortem were included. Pearson’s χ^2^ tests and binary logistic regression analysis were performed.

**Results:**

Alcohol and/or drugs were detected through toxicological analysis for the majority of the total sample (79.5%), which did not differentiate between 15-24-year-old and 25-34-year-old individuals (74.1% and 86.2% respectively). Compared to 25-34-year-old individuals, 15-24-year-old individuals were more likely to engage in suicide by hanging (88.5%). Younger individuals were less likely to die by intentional drug overdose and carbon monoxide poisoning compared to older individuals. Younger individuals who died between Saturday and Monday were more likely to have had alcohol before dying. Substance abuse histories were similar in the two age groups.

**Conclusion:**

Based on this research it is recommended that strategies to reduce substance abuse be applied among 25-34-year-old individuals at risk of suicide. The wide use of hanging in young people should be taken into consideration for future means restriction strategies.

## Introduction

Suicide is one of the leading causes of death worldwide, with approximately 800,000 suicides occurring in 2012 [1]. The problem of suicide is even more pronounced among people aged 15–29, with suicide representing the second leading cause of death and accounting for 8.5% of all deaths in this age group [1].

In Ireland, where the present study was conducted, the most recently available figures on suicide refer to the year 2013, when 487 people died by suicide, representing a rate of 10.6 per 100,000 inhabitants. For young people aged 15–24 years, the suicide rate in that year was 9.9 per 100,000 (males: 16.1 per 100,000, females: 3.5 per 100,000) and for adults aged 25–34 years, the suicide rate was 13 per 100,000 (males: 19.9 per 100,000, females: 6.6 per 100,000) [2]. Corcoran et al. [3] showed a significant increase in suicide in Ireland in recent years, in particular among young males.

### Suicide methods and age

Worldwide, in terms of methods of suicide, hanging is the most common method, with the highest prevalence in Eastern European countries, where up to 90% of men and 80% of women who die by suicide use this means [4]. In some countries, other suicide methods are more prevalent, such as firearms in the United States, Argentina and Switzerland, or poisoning by pesticides in Latin American and Asian countries, especially among women [4]. With regard to young people, in most of the European and the Australasian countries hanging is the most prevalent method among men [5]. Several factors affect the choice of method used, such as availability [6], popularity and socio-cultural context [7]. With regard to possible reasons for the preference for hanging as a method of suicide, Biddle et al. [8] have shown that the main reasons influencing the decision to choose hanging, as reported by those who attempted hanging, are the conviction that this method is rapid, painless and “clean”, not damaging the body or leaving harrowing images for others, together with the accessibility of this method.

Relatively few studies have focused on factors associated with suicide among young people in Ireland, and even fewer studies have addressed differences and similarities between young people and adults. Research into suicide in Ireland should be prioritised due to the increase in suicide rates in recent years, especially among young adults [3].

The present study focuses on some of the acknowledged risk factors for suicide identified in research to date ([5], [9]). A recent report from the World Health Organisation highlights several risk and protective factors for suicide among people in all age groups, including harmful use of alcohol and mental disorders, such as substance use disorder [1].

### Alcohol and other risky behaviours in young people in Ireland

Previous research has shown a link between alcohol use and suicide [10, 11]. Only a limited number of studies investigating completed suicide and alcohol were conducted in Ireland [12, 13], and available studies have not focused on completed suicide in younger individuals. Therefore, research addressing possible associations between suicide method characteristics, toxicological analysis at post mortem, and substance abuse histories in Ireland is needed, especially among young people.

A recent study showed that suicide rates were higher in younger (20–24 years) than older (25–29 years, and 30–34 only in males) age groups living in the South-West region of Ireland [14]. Alcohol misuse is one possible explanation for the higher rates of suicide in younger people. A study by Long and Mongan [15] found that young Irish people aged 18–24 were more likely to engage in binge drinking than adults aged 25–34. A recent cross-sectional study assessed alcohol consumption among university students in Cork city (South-West of Ireland) [16] and showed that the prevalence of hazardous alcohol consumption (excessive use of alcohol) was 65.2% in men and 67.3% in women [16]. It also revealed that 17% of men and 5% of women had more than six units of alcohol at least four times per week and in some cases daily. Binge drinking is associated with a range of problem behaviours, such as accidental death and injury, poor school performance, relationship problems, risky sexual behaviour, suicide and other harmful behaviours [17]. Significant associations between binge drinking and suicidal thoughts and behaviour among students attending second level school and university have been reported in previous research [18, 19]. Aseltine et al. [20] found this association to be stronger for younger adolescents (aged 13 years and younger) compared to older adolescents (aged 14 to 19 years). Young Irish people drink less often than adults, but have more drinks on a single occasion: one recent study reported that 50.6% of young people aged 18–24 years have 9 or more drinks at one time, while only 41.7% of adults aged 25–34 and 28.1% of those aged 35–49 do so [15].

Choice of suicide method is likely to be associated with age. Young people and in particular adolescents with self-regulatory deficits [21], as well as those with high level of impulsivity [22] are more likely to engage in risky behaviours, such as tobacco, alcohol and illicit drug use. Baca-García et al. [23] showed that impulsivity has been associated with greater lethality of suicide methods. Furthermore, access to means of suicide is an important component which influences the choice of method [24, 25]. For young people, particularly adolescents, access to prescribed drugs may be more difficult than for adults.

### Purposes of the study

The present study has been conducted to inform suicide prevention strategies. A greater understanding of the degree of similarities and differences between young people and adults in terms of factors associated with suicide may inform tailored mental health promotion and prevention programmes, particularly where resources are limited [26].

The aim of the study was to identify age-specific socio-demographic characteristics and circumstances of death associated with suicide in Ireland. The overall objective was to compare young people and adults who died by suicide on socio-demographic factors, circumstances of death, suicide methods, substance use histories, and alcohol and drugs detected through coroners’ analysis.

We hypothesised that young people who die by suicide more often employ violent and highly lethal methods and have alcohol in their toxicology at the time of death than adults. We also hypothesised that among young people, a higher proportion of suicides occurs during the weekend compared to adults. In addition, adults would more often use less violent methods and have drugs (both prescribed/over-the-counter and illicit) in their toxicology at the time of death, than young people.

## Materials and Methods

Data were collected between September 2008 and December 2012 on individuals who had died by suicide from May 2007 to June 2012. Data collection was part of the Suicide Support and Information System (SSIS) implemented in Cork City and County by the National Suicide Research Foundation [14, 27]. The SSIS was approved by the Social Research Ethics Subcommittee of the Clinical Research Ethics Committee of the Cork University Teaching Hospitals and the Coroners Society Ireland [14]. The ethics approval concerning the overall project included its dissemination, of which this study is part. None of the authors of this study was a treating physician for any of the subjects who died by suicide. One of the authors (CMA) was directly involved in the collection of the data.

Next of kin were asked to sign a consent form in order to approve the participation of the SSIS study and to use this data for research purposes including the dissemination. Data were de-identified prior to performing the analyses for this research in order to ensure confidentiality and anonymity of individuals participating in the study, as well as those whose deaths were the subject of the research. Digital audio recordings of interviews with family members were saved to the central server as soon as the researchers returned to the office and the audio file on the recorder itself was promptly deleted.

### Sample

Findings are based on data collected on 121 consecutives suicide cases identified by the SSIS: 61 young people (aged 15–24 years) and 60 adults (aged 25–34 years). 115 cases had received a verdict of suicide and 6 cases were classified as open verdicts meeting the case-finding criteria [28]. “Suicide should never be presumed, but must always be based on some evidence that the deceased intended to take his own life”[29]. An open verdict was recorded when the coroner could not indicate that suicidal intent was present *beyond reasonable doubt* [30]. Young people were referred to as those between 15 and 24 years based on previous studies [31–33] and the definition of “youth” by the United Nations [34]. Accurate assessment of homogeneity for this group has been accomplished by conducting a sensitivity analysis, outlined below. Individuals aged 25–34 years were included in the study as we aimed to compare young people to the first decade of adulthood. Within this age range the majority of individuals, especially those who attend university, become independent from their parents, and desire to have their own family [35, 36]. We wanted to examine possible differences between these age ranges. Both of these times might be sensitive periods due to the transitions taking place in the lives of individuals. These transitional periods can raise issues of independence and self-identity. During both periods, individuals might go through identity exploration, self-focus, instability, feeling “in between”, but may also discover new opportunities and experience optimism [37].

### Measures

Details regarding each consecutive case of suicide were obtained via accessing coroners’ records and post-mortem reports [14]. Socio-demographic characteristics examined included date of birth, gender, ethnic origin, nationality, religion, marital status, living arrangement, and employment status. Information was also included on date of death, presence of suicide note, cause of death, history of alcohol and drug abuse and findings from toxicological reports (presence of alcohol and drugs in blood or urine) at time of death. Cause of death was classified in accordance with the International Statistical Classification of Diseases and Related Health Problems 10th Revision [38]. The category involving ‘intentional drug overdose’ included all deaths caused by overdose of prescribed, non-prescribed medication or ethanol, alone or in combination. Prescribed drugs included anti-depressant, lithium, benzodiazepines, paracetamol, insulin, anti-psychotic drug, salicylate, methadone, and other drug, and non-prescribed included opiates such as heroine and morphine, non-opiates such as cocaine and ecstasy, and other drugs.

Histories of alcohol and drug abuse were obtained from the semi-structured psychological autopsy interviews (e.g. “*Was there a personal (with regard to the deceased) history of substance abuse?”)*, which were conducted with family members by senior research psychologists using a standardised protocol [39]. Health care professionals were asked to complete a questionnaire relating to the person’s health and treatment history. The section exploring the deceased’s use of alcohol and drugs included the following questions: “*Did the deceased have a history of alcohol or drug abuse?”*, *“Had the deceased made any recent attempts (in the year prior to death) to stop abusing alcohol or drugs?”*, *“Was there a recent increase in the deceased’s abuse of alcohol or drugs?”*, *“Was there any evidence that the deceased had been drinking or taking drugs at the time of death?”*.

### Statistical analysis

We conducted a sensitivity analysis (Pearson’s χ^2^ tests) separating adolescents aged 15–19 years and people aged 20–24 years to ensure that these groups were similar and it was reasonable to collapse them. We included the following variables: standard socio-demographic characteristics, characteristics of the suicidal act, presence of a suicide note, additional methods, alcohol and drug histories, and toxicology, alcohol and drugs. No differences were identified between these two groups, with the exception of the employment status. Full-time student was the most common professional status among adolescents, whereas both unemployed and being a paid employee were the most common status among people aged 20–24 years.

Univariate analyses—performing Pearson’s χ^2^ tests—were conducted separately for each variable to determine whether there were any differences between young people and adults. These analyses aimed to estimate the possible association of the variables shown below with age. The variables included were standard socio-demographic characteristics, characteristics of the suicidal act, presence of a suicide note, additional methods, alcohol and drug histories, and toxicology, alcohol and drugs. Drugs were included as a general category (presence and absence of any drugs in toxicology). Additionally, we included different drug categories: antidepressants, benzodiazepines, opiates and other drugs. Drugs were grouped in these four categories as they were the most commonly used drugs in the sample.

Information on alcohol and drug history was available for 58% of the sample (n = 70, 31 young people and 39 adults). Information on toxicology was available for 96.7% of the whole sample (n = 116; n = 58 young people, and n = 58 adults). Information on suicide notes was available for 85.1% of the sample (n = 103; n = 53 young people, and n = 50 adults).

A binary logistic regression analysis was conducted to identify factors associated with young age. Based on the results of the univariate analysis, the variables included in the analysis were as follows: day of death (Monday, Tuesday, Wednesday, Thursday, Friday, Saturday, Sunday); marital status (single, married/cohabiting, widowed, divorced/separated); living arrangement (alone, with family of origin, with partner/children, other); employment status (unemployed, unemployed due to disability, employed/full-time student/housewife/retired); benzodiazepines found in toxicology (presence, absence); and method of suicide (hanging, self-poisoning, other).

The ‘forward selection method’ was used as the variable selection technique in the logistic regression model. The criterion for entering into the model was 5% and the criterion for exclusion from the model was set at 10% level of significance. In all cases, odds ratios (OR) were reported.

The Hosmer and Lemeshow Test was used to test the goodness-of-fit, with a cut-off p-value greater than 0.05 to indicate goodness of fit. All analyses were performed using IBM SPSS 20 [40].

## Results

The total sample comprised of 121 consecutive cases: most cases involved males (83.5%; 101/121), of Irish nationality (93.3%; 112/120), Catholic religion (94.2%; 98/104), single (75.8%; 91/120) who were living in a house or flat (75.21%; 91/121) (Table 1).

**Table 1:** Socio-demographic characteristics by age group.

** **	**Young people**	**Adults**	
	15–24 years	25–34 years	
	(n = 61)	(n = 60)	
	%	%	
**Gender**			
Male	88.5	78.3	*p* > 0.05
Female	11.5	21.7	
** **	100	100	
**Ethnic origin**			
White	98.4	96.7	*p* > 0.05
Other	1.6	1.3	
	100	100	
**Nationality**			
Irish	98.4	86.7	*p* > 0.05
Other European	1.6	8.3	
Other	-	3.3	
** **	100	98.3*	
**Religion**			
Catholic	80.4	81.7	*p* > 0.05
Atheist	1.6	3.3	
Protestant	1.6	-	
Buddhist	1.6	-	
Hindu	-	1.7	
** **	85.4*	86.7*	
**Marital status**			
Single	90.2	60	*p* = 0.001
Married	9.8	30	
Divorced/Separated	-	8.3	
** **	100	98.3*	
**Living arrangements**			
Alone	3.3	16.7	*p* = 0.0002
With family of origin	73.8	30	
With partner and/or children	9.8	31.7	
Other shared	13.10	21.6	
	100	100	
**Employment status**			
Employed/Self-employed	27.9	41.7	*p* = 0.004
Unemployed	37.7	35	
Full-time student	24.6	3.3	
Long term disability	1.6	6.7	
** **	91.8*	86.7*	

*Missing values ranged between 1 and 17.

The comparison of young people who died by suicide versus adults on socio-demographic characteristics revealed that the majority of young people lived with their family of origin (73.8%), whereas less than one-third of the adults lived with their family of origin (30%), and a partner (31.7%) respectively. The two groups also differed in marital status (90% of young people were single compared to 60% of adults) and employment status (27.9% of young people were employed/self-employed compared to 41.7% of adults). The two groups were similar in terms of gender, nationality, ethnicity and religion.

Among young people, the majority of suicide deaths (n = 54; 88.5%) involved hanging, and the remainder (n = 7; 11.5%) involved other methods.

Among adults, hanging was the most common method of suicide (n = 40; 66.7%). However, self-poisoning (intentional drug overdose n = 9 and carbon monoxide poisoning n = 3) was more common among adults (n = 12; 20%) than young people (n = 3; 4.9%). Intentional drug overdose referred to illicit drug overdose, overdose of medication and ethanol overdose, alone or in combination (a list of the drugs was outlined in section *Materials and Method*). Other methods (n = 8; 13.3%) involved drowning, fire-arms, and jumping from height.

Findings revealed that young people die by hanging more often than adults (OR = 3.86; CI 95% = 1.49–10.00; *p* = 0.004). A separate analysis examining self-poisoning as the outcome showed that young people die less often using this method than adults (OR = 0.21; CI 95% = 0.06–0.78; *p* = 0.012).

There was no significant difference between young people and adults with regard to history of alcohol and drug abuse (74.2%, 61.5% respectively) (χ^2^ = 3.73; *p* >0.05). History of alcohol and drug abuse was available for 58% of the total sample (n = 70, 31 young people and 39 adults). Based on toxicological analysis of samples taken at post-mortem, 79.5% of the total sample had used either alcohol or drugs at the time of death. For 70.1% of all individuals, drugs and/or alcohol were combined with other methods. Young people and adults who died by other methods than intentional drug overdose, were not significantly different in terms of alcohol or drugs (74.1% and 86.2% respectively).

A similar proportion of young people and adults had “alcohol only” (24.1% in both age groups) and “alcohol and drugs” (27.6% and 29.3% respectively) in their toxicology. Even though adults more often had “drugs only” in their toxicology (32.8%) than young people (24.1%), this was not statistically significant. Nearly twice as many adults than young people had used benzodiazepines, based on the coronial information (40.0%, 24.5% respectively) (χ^2^ = 2.82; *p* = 0.093).

In terms of the presence of a suicide note, a similar proportion of young people (36.2%) and adults (35.0%) left a suicide note at the time of death (χ^2^ = 0.02; *p* = 0.891).

**Fig 1:**
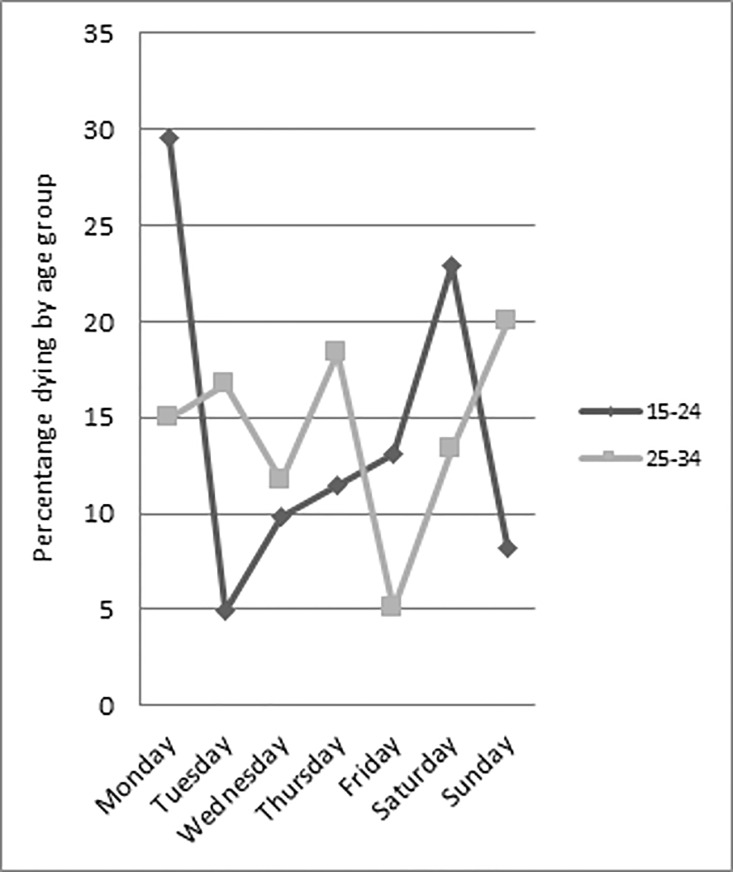
Suicide and day of the week by age group.

Comparing young people and adults, there was significant variation in the day of the week on which the suicide occurred (χ^2^ = 14.52; *p* = 0.024) (Fig 1). A total of 60.0% of young people died between Saturday and Monday, with a peak on Monday (29.5%). Conversely, the highest proportion of suicides among adults occurred on a Sunday (20.0%).

Overall, individuals who died at the weekend had alcohol detected through toxicological analysis of blood and urine samples taken at post mortem (62.2% versus 37.8%) more often than those who died mid-week, however, this was not statistically significant. Compared with suicides occurring over the weekend, alcohol was detected less often among those who died between Monday and Friday (47.5% versus 52.5%). An analysis was conducted separately for young people and adults on the association between day of the week and alcohol in toxicology. In young people who died at the weekend, alcohol was detected for 66.7% compared to 43.9% of those who died between Monday and Friday. Among adults, the proportion was similar for those with alcohol detected in their system who died at weekend and those who died between Monday and Friday (57.9% and 51.3% respectively). A trend towards significance was observed among young people: those who died at the weekend had alcohol in their toxicology more often than those who died during the week (χ^2^ = 2.88; *p* = 0.089).

The final logistic regression analysis illustrated that factors significantly associated with young age included suicide by means of hanging (Ref. hanging; OR = 7.05; CI 95% = 1.30–38.23; *p* = 0.023) and living with the family of origin (Ref. living alone; OR = 3.56; CI 95% = 1.01–12.55; *p =* 0.049) (Hosmer-Lemeshow Test = 0.96; Nagelkerke R Square = 0.31). This result was consistent with the univariate analyses.

Most of the individuals who died by hanging had taken alcohol or drugs or both substances, specifically 71.1% of young people and 86.8% of adults. Even though this was higher among adults, this difference was not significant (χ^2^ = 5.12; *p =* 0.16). The main suicide methods and concurrent alcohol and drug use for both age groups are shown in the Table 2.

**Table 2:** Alcohol and drugs in toxicology by age group.

	**Cause of Death**	**Total**	**Alcohol Only**	**Alcohol and Drugs**
**Young people^♣^ (n = 58)**	Self-poisoning (intentional drug overdose and carbon monoxide poisoning)	3	3(2)*	-
	Hanging	52	23	14
	Other highly lethal methods (fire-arms, cutting/stabbing, jumping/lying before train)	3	1	2
**Adults^♣^ (n = 58)**	Self-poisoning (intentional drug overdose and carbon monoxide poisoning)	12	6(5)*	6(4)*
	Hanging	38	24	9
	Other highly lethal methods (fire-arms, jumping from a height, drowning)	8	3	2

* The main cause of death was drug overdose.

^♣^ Information on toxicology was not available for 5 suicide cases (young people = 3, adults = 2)

## Discussion

This is one of the first studies in Ireland comparing young people and adults on socio-demographic factors, method characteristics and substance abuse histories associated with suicide. Young people and adults differed on suicide methods, living arrangement, day of the week in which they took their life, and specific drugs (benzodiazepine) detected through toxicological analysis.

Even though hanging was the most common method of suicide overall, it was significantly more common among young people compared to adults. Adults died significantly more often by self-poisoning (intentional drug overdose and carbon monoxide poisoning), than young people. This outcome is consistent with previous research, indicating that the risk of suicide by hanging decreased with age [41]. Some studies [41–43] have shown that the risk of suicide by firearms increased with age; however, we cannot verify that result due to the small number of people who had used this method in the current sample.

Hanging is one of the most lethal methods of suicide and cannot be easily addressed with preventive actions. While some settings (e.g. farms, industrial areas, trade workshops) have a greater availability of materials which could be used for hanging, theoretically, any material can be used as a method of hanging [44]. For this reason, restricting and reducing suicide by hanging is difficult and signifies a major public health challenge. Nevertheless, urgent actions are needed [1]. One possibility might be to try to reduce the “cognitive availability” of hanging by improving media portrayals and reporting of suicidal behaviour [45]. Cognitive availability of hanging is represented in one’s individual awareness of this suicide method as an option and the knowledge of the characteristics of this method, such as accessibility, painfulness, and outcome [46]. Cognitive availability might therefore play an important role in the choice of suicide method. This is important to take into account as methods with high lethality rates, particularly hanging, may become more popular. The choice of a method, such as hanging, rather than another method, can have a significant impact on the outcome of an attempt [45]. The two most common sources of information about methods of suicide are the media and experiential knowledge gathered during an individual’s past experience [45]. The role of the media, including television, film, new stories, and the Internet (e.g. introducing new methods, suggesting implementation of these methods) is relevant in influencing the choice of suicide method [45]. Recent studies showed that choice of suicide method is more easily influenced in younger adults [47], and that they are more likely to be influenced by imitation effects [48].

Our findings showed that the majority of individuals in both age groups were living either with their family of origin or their partner. Before a suicide, it is often difficult for family members to broach the topic of suicide with an at-risk loved one [49], but family members may be an important resource in terms of identifying risk and encouraging help-seeking [50].

The present study also revealed the rate of alcohol by toxicological analysis of blood and urine samples taken at post mortem (overall rate 52.1%; in men 46.2%, in women 35%) among young people and adults, which is in line with a previous systematic review [51] that identified a range of 10–69% of alcohol present in the toxicology of people who had died by suicide.

Results from the present study did not reveal a significant association between alcohol and age among those who died by suicide—young people and adults were similar in terms of alcohol use at time of death—nor a significant association between alcohol and method used. Nevertheless, overall, approximately 50% of individuals who died by suicide had alcohol detected at the time of death. This finding can contribute to informing governments and policy makers about suicide prevention efforts. Our results found that more than 70% of young people and more than 60% of adults had a history of alcohol and/or drug abuse.

This study reveals that young people who died by suicide were more likely to die by hanging and between Saturday and Monday. Hanging is considered one of the most lethal methods of suicide, which is often linked to impulsivity [52]. Alcohol consumption among young people is more likely to occur during weekends [53, 54] and drinking may exacerbate depression and depressive symptoms [55]. Even though alcohol use is not the only risk factor for suicide, it increases disinhibiting thoughts and behaviour [11] and depressive symptoms [55], impacting on the other risk factors for suicide [11]. Therefore, alcohol use may increase the likelihood that suicidal behaviour will occur.

### Strengths and limitations

The present study has both strengths and limitations. A major strength is that this is one of the first studies in Ireland involving consecutive cases of suicide involving young people, and representing a reasonably-sized sample of the young and adult population subgroups. The information on alcohol and drug histories comes from the psychological autopsy interviews which were standardised according to the recent research on psychological autopsy [56], representing a further strength. These interviews were conducted only for a subset of the sample, where alcohol and drug histories were only available for 58% of the sample (n = 70, 31 young people and 39 adults). Information on toxicology was not available for 5 suicide cases (3 young people and 2 adults). Additionally, for some of the socio-demographic characteristics, the missing values ranged between 1 and 17.

While the statistical power was limited due to the relatively small sample size, the present study covers one of the larger samples when it concerns suicide among young people in Ireland. Further limitations are the absence of a general population control group, and the levels of alcohol in terms of blood alcohol concentration and amount of drugs, in particular concentration of drugs in blood, tissue or urine detected from post-mortem examination. As we did not assess the levels of alcohol and drug intoxication, we were not able to estimate the extent to which these substances influenced the behaviour of the individuals.

Furthermore, the therapeutic and recreational drugs were collapsed together due to the relatively small numbers. Therefore, we were not able to discern whether there were differences in outcomes as a function of specific types of drugs. The lack of information on differences between therapeutic and recreational drugs represents a limitation, as this information has important implications for intervention targeting.

## Conclusion and implications for suicide prevention

This study involves a comparison between age groups, which will be of value for targeted suicide prevention strategies. The research outcomes showed that among young people, alcohol use was more common among those who died between Saturday and Monday than young people who died during the other days of the week. Among young people, hanging was the most prevalent method, whereas among adults, both hanging and intentional drug overdose were prevalent.

Strategies addressing known factors for suicide should include “universal” prevention strategies, whereby the entire population should be reached [1]. Important examples of universal strategies include restrictions of access to means for suicide, and the promotion of responsible media reporting [1].

Suicide prevention awareness and skills training for primary health care workers [57]—including general practitioners and pharmacists—should take note of the findings of this study, on cause of death, alcohol and drug use among people aged 15–34 years, taking into account similarities and differences between young people (15–24 years old) and adults (25–34 years old). Based on our findings, the psychosocial assessment by the mental health services of individuals at-risk of suicide—e.g. those who have attempted suicide—should always include the assessment of drug use (prescribed and illicit drugs). Suicide prevention interventions aimed at those who are at risk of suicide, should also take into account drug use when taken as part of a suicide attempt.

Programmes promoting positive mental health in education settings [57] aim to increase emotional competence and personal skills in order to cope with personal difficulties and stress. In addition, these programs could specifically highlight how the use of alcohol and illicit drugs might increase personal difficulties, addressing the common myth that they help to reduce stress.

There are many factors behind the choice of suicide method, and the socio-cultural acceptability of the methods represents one of them [58]. Strategies for suicide prevention should include actions to reduce the cognitive availability of hanging, particularly among young people. This could be achieved by closer collaboration with the media when reporting cases of suicide by hanging. In general, the media should decrease the representation of fictional suicides by hanging and suicide reporting involving this method. In accordance with media guidelines for reporting of suicide, the media should avoid explicit detail of the event, inappropriate use of language (e.g. epidemic), and sensationalism [59, 60]. The media should also avoid showing pictures, stills or video content of the scene of a suicide by hanging. However, restricting the media, specifically the Internet, would be an arduous task. The challenge should therefore be to deliver information of content in the way that most of people would be dissuaded from using hanging as a suicide method.

Since hanging was the most common suicide method in young people, it is essential that this area of research is pursued and developed. In particular, research examining psychological factors associated with young people engaging in highly lethal methods, such as hanging, should be prioritised.
